# Whole genome sequencing of the pulmonary microbiome in interstitial lung disease subtypes

**DOI:** 10.1186/s12931-025-03404-5

**Published:** 2025-11-19

**Authors:** Kristel S. Knudsen, Gunnar Husebø, Rune Nielsen, Andreu Paytuvi-Gallart, Roberto Malinverni, Walter Sanseverino, Sverre Lehmann, Tomas M. Eagan

**Affiliations:** 1 Department of Clinical Science, University of Bergen, Jonas Lies vei 87, Bergen, 5021 Norway; 2 Department of Thoracic Medicine, Haukeland University Hospital, Bergen, Norway; 3 Sequentia Biotech SL, Barcelona, Spain

**Keywords:** Microbiome, Interstitial lung disease, Whole genome sequencing

## Abstract

**Background:**

Interstitial lung diseases (ILDs) represent a heterogeneous group of lung disorders, some of which remain unclassifiable. The pulmonary microbiome may contribute to ILD pathogenesis, yet research is limited. Whole genome sequencing (WGS) offers enhanced microbial characterization. Here we evaluate the dysbiosis index (DI) as a potential biomarker to refine the classification of unclassifiable ILD.

**Methods:**

Protected bronchoalveolar lavage (PBAL) samples were collected from the right middle lobe of 12 IPF patients, 34 sarcoidosis patients, 11 unclassifiable ILD patients and 100 healthy controls. WGS was performed with the Illumina NovaSeq platform. Operational Taxonomic Units (OTU) were identified with GAIA 2.0 software, and statistical analyses were performed in R. The DI was calculated based on differential abundant species.

**Results:**

Alpha diversity was significantly higher in IPF and sarcoidosis patients compared to healthy controls. Beta diversity analysis revealed distinct microbial composition in IPF, sarcoidosis and unclassifiable ILD groups relative to controls. Differential abundance analysis identified several taxa with significant variation across groups. Notably, the dysbiosis index demonstrated high sensitivity and specificity in distinguishing IPF and sarcoidosis from healthy controls and provided further insight into the microbial characterization of unclassifiable ILD.

**Conclusions:**

The pulmonary microbiome in unclassifiable ILD patients differed from healthy controls, and the dysbiosis index may provide exploratory insights for future ILD characterization.

**Supplementary Information:**

The online version contains supplementary material available at 10.1186/s12931-025-03404-5.

## Introduction

Interstitial lung diseases (ILDs) are a heterogeneous group of disorders characterized by impaired lung function and varying degrees of inflammation and fibrosis in the lungs [1]. Some ILDs are associated with an underlying systemic condition, while others are without a known trigger and referred to as idiopathic [2]. ILDs are usually classified by multidisciplinary teams according to the latest international guidelines [3–7]. Some ILDs cannot be classified as a result of either inadequate clinical, radiologic or pathologic data or as a consequence of shortcomings of our current classification system [8–10]. Newer approaches in classification and treatment of ILDs with precision medicine are emerging [11].

Two of the most common ILDs are idiopathic pulmonary fibrosis (IPF) and the granulomatous disease sarcoidosis. New culture independent techniques with next generation sequencing have revolutionized our understanding of resident microbiomes and the healthy lung is no longer considered sterile [11–13]. Recent research has shown that both IPF [14–16] and sarcoidosis [17–21] have different pulmonary microbiome profiles compared with other chronic pulmonary disorders and healthy controls [22]. An imbalance in the pulmonary microbiome can be considered as an indicator of a given disease, and the term ”dysbiosis” is often preferred. Several dysbiosis indices have been developed [23] and these indices hold potential as biomarkers for disease processes. If shown to be distinct for specific diseases, they may also facilitate disease classification. However, this hypothesis remains untested for the pulmonary microbiome.

Most of the microbiome studies to date have used the technology of 16 S rRNA sequencing [24], but whole genome sequencing (WGS) is now increasing in volume and scope due to rapidly dropping sequencing costs [25]. By targeting the whole genome, WGS allows for better species level characterization, which has often been unreliable below the genus level with 16 S rRNA sequencing [26–29]. To our knowledge, no published studies have explored the differentiation of the pulmonary microbiome across various ILDs using WGS, and only a few WGS studies have examined the microbiome in pulmonary diseases [30–36].

In this study, we used WGS of protected bronchoalveolar lavage (PBAL) samples to compare the pulmonary microbiome across patients with IPF, sarcoidosis, unclassifiable ILD, and healthy controls. Our objective was to calculate the dysbiosis index and evaluate its potential as a biomarker for characterizing unclassifiable ILD.

## Methods

### Study design

Subjects were recruited from the Bergen ILD Microbiome Study (“MicroILD”) and the Bergen COPD Microbiome Study (“MicroCOPD”), with data collected between 2012 and 2016 at our outpatient clinic located within the Department of Thoracic Medicine, Haukeland University Hospital [15, 20, 37, 38]. The Norwegian regional ethical committee (REK) approved the studies with case numbers 2014/1393 & 2011/1307 respectively. Written and oral consent were obtained from all participants.

From the MicroILD study, we included 12 IPF patients, 34 stable sarcoidosis patients, and 11 unclassifiable ILD patients. The sarcoidosis patients were previously diagnosed and followed in a stable condition at our outpatient clinic [17]. The IPF [12] and unclassifiable ILD patients were included among patients suspected of ILD before bronchoscopy. Diagnoses were set at multidisciplinary team meetings according to the 2011 guidelines [39]. For most of the IPF and unclassifiable ILD patients, lung biopsies were contraindicated or deemed unnecessary for the diagnosis. The IPF patients had a medical history consistent with IPF, no sign of auto-immunity, and a pattern on high resolution computed tomography (HRCT) consistent with possible usual interstitial pneumonia (UIP) (*n* = 3) or UIP (*n* = 9). Surgical biopsies were performed in the possible UIP patients and showed a UIP pattern for all three patients.

The 100 healthy controls were recruited from the MicroCOPD study. Healthy controls could neither have any previous lung disease, nor findings indicative of lung disease upon inclusion. Other exclusion criteria in the MicroCOPD study included double platelet inhibition, cardiac valve prosthesis, and acute coronary syndrome the preceding 6 weeks [34].

All subjects were in a stable condition and examined with consistent methodology in one centre by the same investigators. Collection of metadata involved obtaining blood samples, measuring lung function, and conducting a standardised interview covering medical history, comorbidities and medications. A comparison of comorbidities and medication use between study categories is provided in Supplementary Table 1, whereas a more detailed description of the sarcoidosis patients is provided in Supplementary Table 2. A description of the HR CT findings for the unclassifiable ILD patients is given in Supplementary Table 3.

### Bronchoscopy

All participants were voluntarily examined by bronchoscopy, and details have been previously published [15, 20, 37, 38]. Briefly, the procedure was performed with oral access and the participants were awake in a supine position. Locally lidocaine and a small dosage light sedation of intravenous alfentanil were offered to all. To minimize contamination, we refrained from suctioning before reaching the carina. We sampled 3 × 50 ml PBAL (2 × 50 ml; healthy controls) through a sterile protective sheath from the right middle lobe.

### Whole genome sequencing

Pre-sequencing procedures involved extraction of bacterial DNA through a combination of enzymatic and mechanical lysis, using MP Biomedical`s FastPrep-24 instrument and the FastDNA Spin Kit from MP Biomedicals (LLC, Solon, OH, USA).

The CeleroTM DNA-Seq Library Preparation Kit (Tecan, Männedorf, CH, Switzerland) was used for library.

preparation. Quantification of the input DNA and final libraries was conducted using Qubit 2.0 Fluorometer (Invitrogen, Carlsbad, CA, USA) and quality assessment was performed with the Agilent 2100 Bioanalyzer using High Sensitivity DNA assay (Agilent technologies, Santa Clara, CA, USA).

Sequencing was performed on the Illumina NovaSeq 6000 platform in paired-end 150 bp mode.

### Bioinformatic analyses

Raw reads were analysed with GAIA (v2.02) ((https://metagenomics.sequentiabiotech.com) to generate operational taxonomic unit (OTU) tables at different taxonomic levels [40]. GAIA is a software pipeline that starts with platform-specific quality control, applying read trimming and filtering based on sequencing technology. It then performs taxonomic classification using a mapping-based approach, retrieving the best alignments and applying a Lowest Common Ancestor (LCA) algorithm. The classification is based on a custom database derived from the NCBI BLAST “nt” database [41]. To filter potential false positives, only species supported by at least two reads in at least two samples were included in the downstream analyses. Human DNA sequences were removed prior to bacterial OTU identification by removing all taxa under the phylum *Chordata*.

Statistical analyses were performed in R (v4.4.2) with RStudio. Taxonomic analyses were conducted using the Mia (v1.6.0) and Phyloseq (v1.42.0) packages. Alpha diversity was assessed on rarefied data, including the Observed, Chao1, Shannon and Gini-Simpson metrics. Beta diversity was evaluated using Bray-Curtis dissimilarity between all study participants and visualised with Principal Coordinates Analysis (PCoA). Statistical differences between groups were assessed using PERMANOVA, adjusted for age, sex, and smoking status.

Differential abundance was identified using DESeq2 (v1.38.3) [42] based on the negative binomial distribution. CLR (Centered Log Ratio) normalization values were calculated using the R package mia [43]. Log2 fold change values were visualised in volcano plots, with a corrected p-value of 0.05 set as the threshold for significance.

The dysbiosis index (DI) is a composite metric that quantifies how far a microbial community’s composition deviates from a reference community. In this study we computed a taxon-based DI, using species identified as differentially abundant with DESeq2 for either IPF versus controls or sarcoidosis versus controls. For each contrast, DI was calculated following the approach of Xia et al. ^44^. For discrimination, the optimal cut-point was determined using Youden’s J, which balances sensitivity and specificity. Unclassifiable ILD samples were subsequently evaluated against both indices to identify whether their microbial composition aligned more closely with an IPF-like or sarcoidosis-like profile.

Demographic data were analysed in Stata, where categorical variables were presented as proportions and continuous variables as means or medians depending on the distribution. Differences in sex and smoking were tested with Pearson chi square test. Differences in age, lung function and BAL cell content were tested with Kruskal Wallis tests. A p-value < 0.05 was considered statistically significant in all analyses.

## Results

The demographics of the participants are shown in Table 1. Both IPF and unclassifiable ILD patients had reduced forced vital capacity (FVC) and higher neutrophil counts in PBAL compared to sarcoidosis patients and healthy controls. As expected, patients with sarcoidosis were younger, had smoked less, and had elevated lymphocyte counts in PBAL.

**Table 1:** Characteristics of the study population

Variables	Controls	IPF	Sarcoidosis	Unclassifiable ILD	Statistically significant differences
	*n* = 100	*n* = 12	*n* = 34	*n* = 11	*p* < 0.05
*Sex*,* %*					ll
Women	43	33	24	55	
Male	57	67	76	45	
*Age*,* median years (IQR)*	66.9 (60.2–70.2	73.7 (66.1–81.8)	57.7 (45.8–63.7)	63.7 (52.9–76.0)	‡,§, ll
*Pulmonary function in % predicted*,* median (IQR)*					
FEV_1_	105 (95–114)	76 (67–83)	87 (77–98)	69 (54–78)	*, †, ‡,§, ll
FVC	114 (103–122)	72 (63–87)	98 (89–104)	73 (53–81)	*, †, ‡,§, ll
DLCO	96 (85–107)	54 (42–70)	86 (80–95)	62 (34–70)	*, †, ‡,§, ll
*Smoking habits*,* %*					
Current	24	9	6	9	ll
Ex	58	58	44	64	
Never	18	33	50	27	
*BAL cell counts %*,* median (IQR)*					
Macrophages	83.1 (78.8–91.5)	76.4 (66.0–90.0)	76.4 (62.7–86.5)	78.0 (40.0–90.5.0.5)	†,ll
Neutrophils	2.9 (1.6–5.5)	6.5 (3.5–21.5)	0.9 (0.0–2.1.0.1)	12.0 (6.5–30.0)	*, †, ‡,§, ll
Lymphocytes	9.1 (5.3–15.9)	6.6 (4.7–12.0)	16.3 (8.2–30.9)	18.6 (7.0–21.3.0.3)	ll
Eosinophils	0.3 (0.0–1.3.0.3)	5.0 (5.0–5.0)	0.7 (0.3–1.6)	3.4 (3.4–3.4)	ll

Differences in sex and smoking tested with Pearson chi square test. Differences in age, lung function and BAL cell content tested with Kruskal Wallis test

* Unclassifiable ILD vs. Sarcoidosis

† Unclassifiable ILD vs. Controls

‡ IPF vs. Sarcoidosis

§ IPF vs. Controls

ll Sarcoidosis vs. Controls

### Diversity

**Fig. 1:**
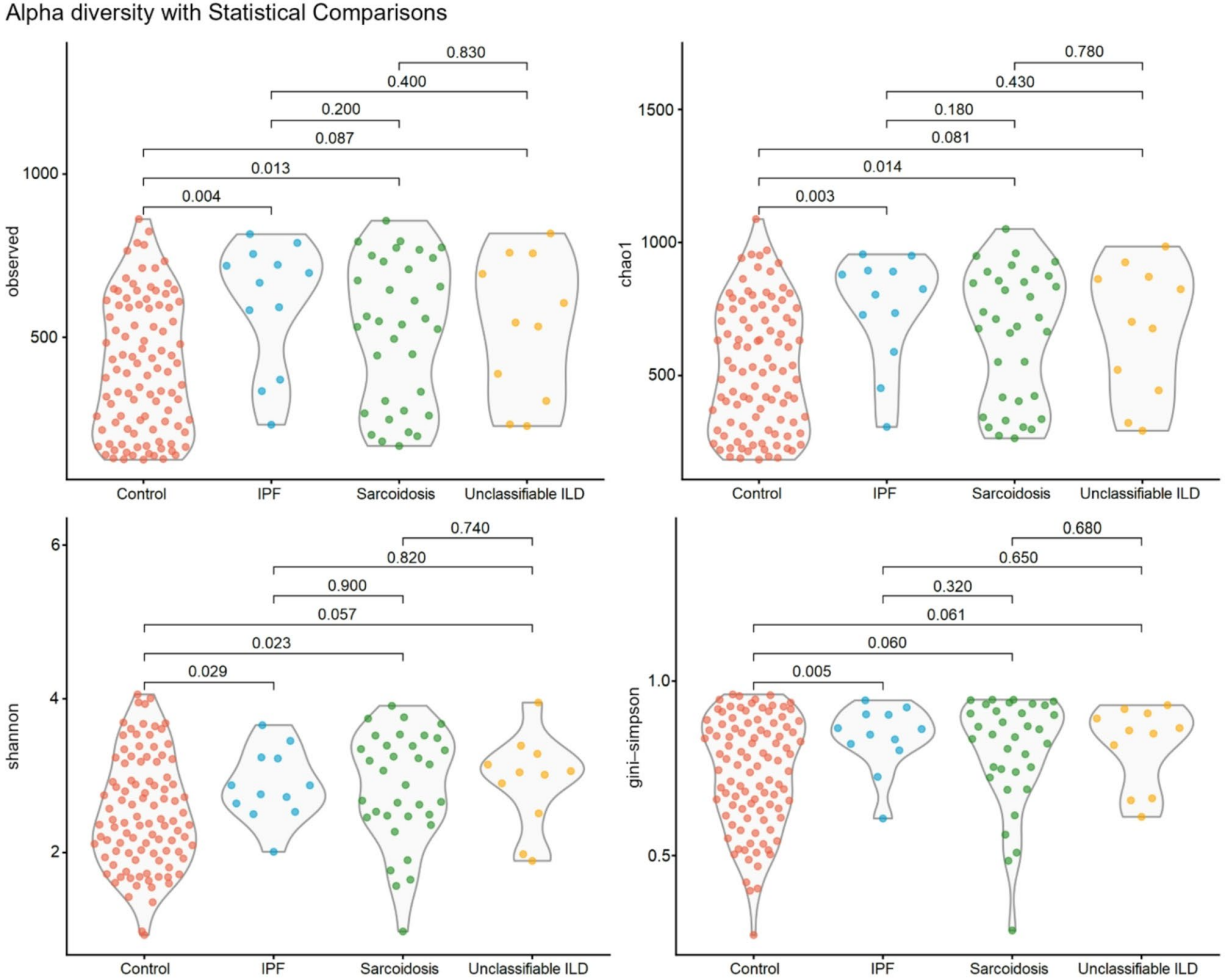
Alpha diversity with statistical comparisons across different study groups. Control (red), IPF (blue), sarcoidosis (green), and unclassifiable ILD (yellow). The indices tested are Observed (top left), Chao1 index (top right), Shannon (bottom left) and Gini-Simpson (bottom right). Each dot represents an individual sample, while the violin plots display the distribution of data within each group. P-values indicate statistical significance for differences between groups

The within sample alpha diversity for each study category is illustrated using violin plots in Fig. 1. The IPF patients had significantly higher alpha diversity than controls with the Observed (*p* < 0.01), Chao1 (*p* < 0.01), Shannon (*p* = 0.02) and Gini-Simpson (*p* < 0.01) indices. The alpha diversity was significantly higher in sarcoidosis compared with controls for the indices Observed (*p* = 0.01), Chao1 (*p* = 0.01) and Shannon (*p* = 0.02). The unclassifiable ILD patients showed no significant differences compared to controls. There were no statistically significant differences in alpha diversity between IPF, sarcoidosis and unclassifiable ILD (Fig. 1).

**Fig. 2:**
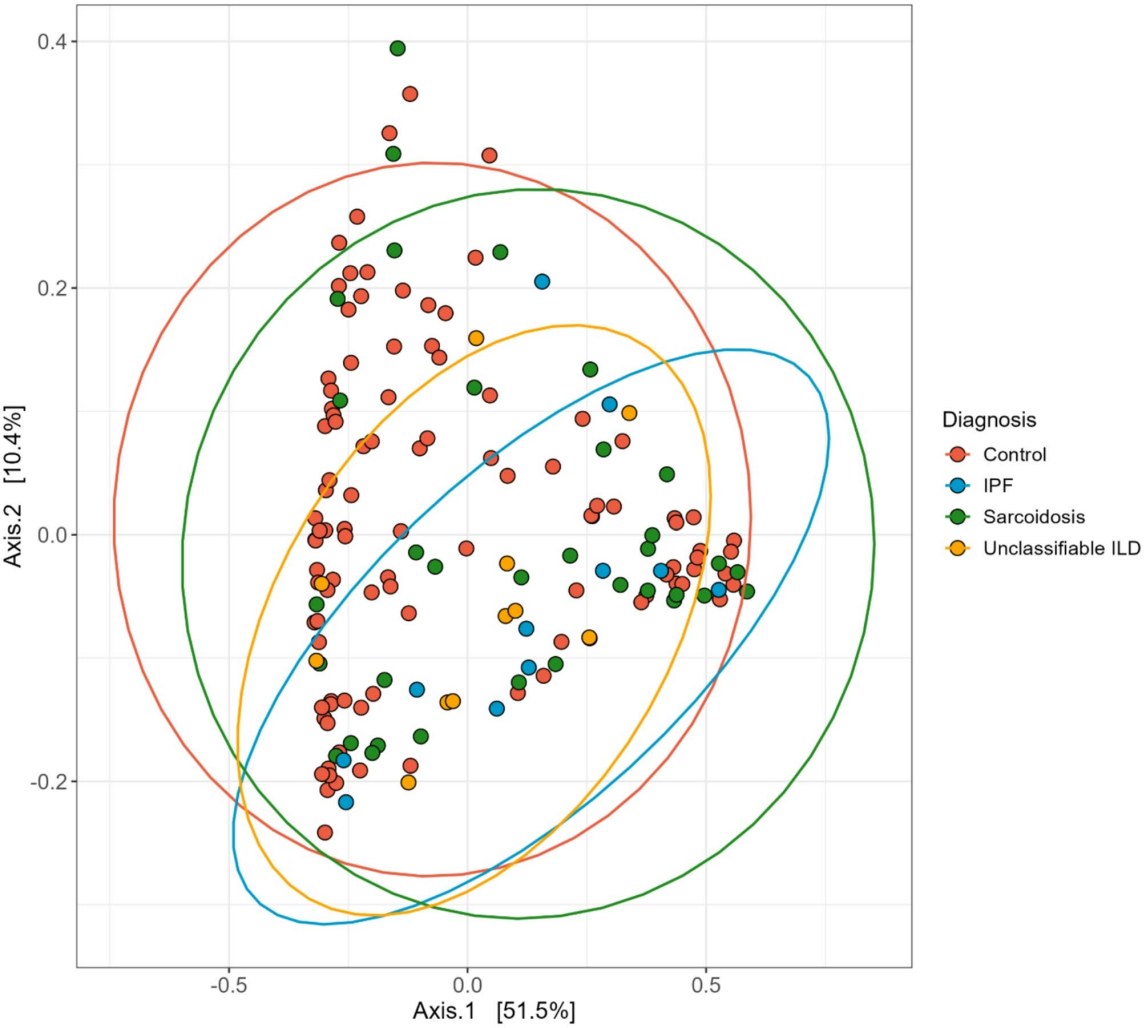
Principle Coordinate Analysis (PCoA) plot by study groups based on Bray-Curtis dissimilarity. Control (red), IPF (blue), sarcoidosis (green) and unclassifiable ILD (yellow). Each point represents an individual sample, and the ellipses indicate 95% confidence intervals for group clustering. Axis 1 (52.6%) and Axis 2 (10.6%) represent the percentage of variation explained by the respective principal coordinates

The beta diversity is displayed with a Principal Coordinates Analysis (PCoA) plot in Fig. 2. The plot illustrates the clustering patterns among the different study groups control (red), IPF (blue), sarcoidosis (green) and unclassifiable ILD (yellow). From the plot, IPF and unclassifiable ILD appear to cluster more closely on one hand, and controls and sarcoidosis on the other hand.

The control group had significantly different beta diversity compared to IPF (*p* < 0.01), sarcoidosis (*p* < 0.01) and unclassifiable ILD (*p* = 0.03). There were no statistical differences in beta diversity between IPF, sarcoidosis and unclassifiable ILD.

### Taxonomy

**Fig. 3:**
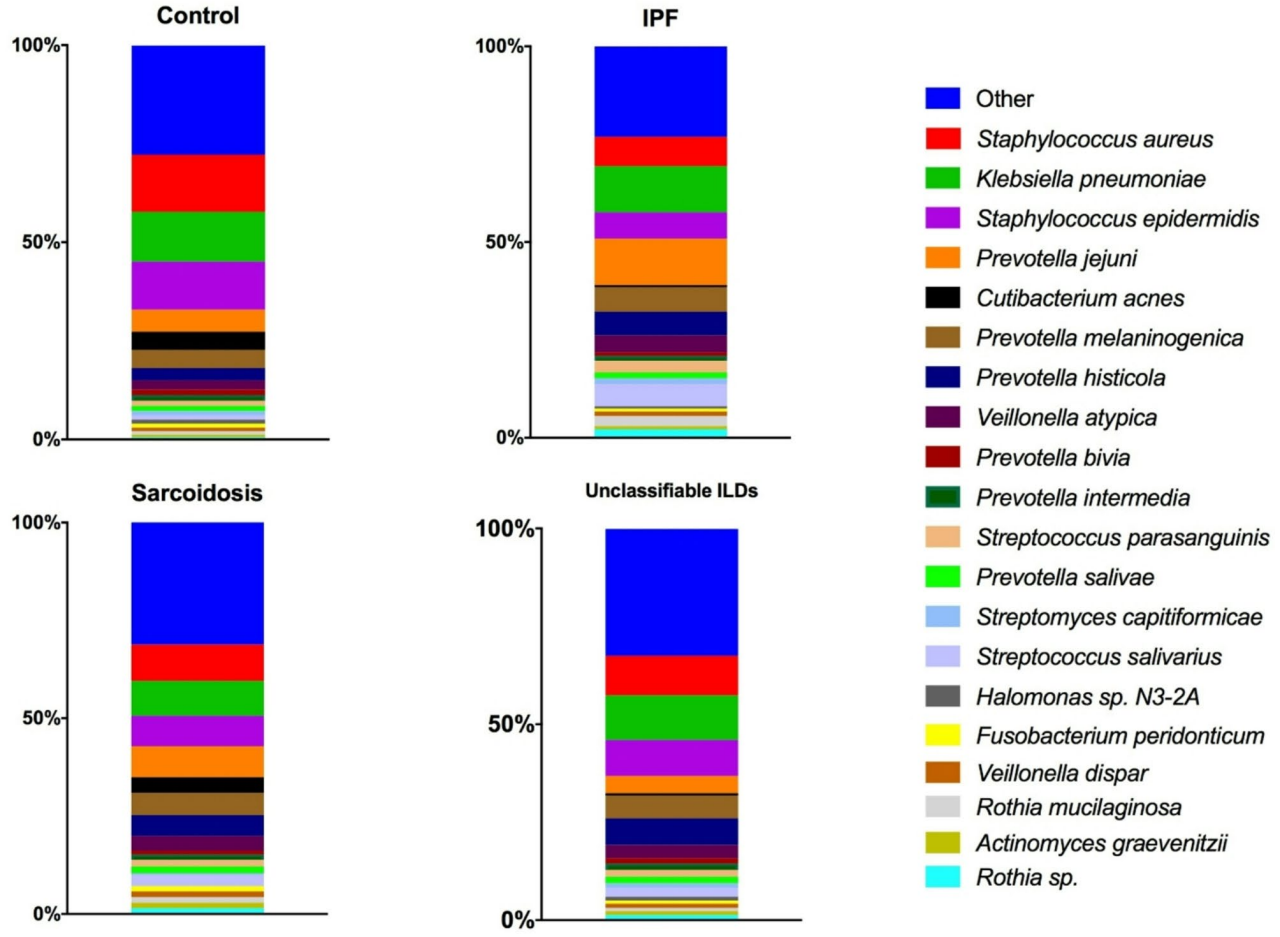
Bacterial taxonomy at species level by study groups The stacked bar plot illustrates the relative abundance of species across the study groups: control, IPF (idiopathic pulmonary fibrosis), sarcoidosis and unclassifiable ILD

Figure 3 illustrates the relative abundance of the 20 most prevalent bacterial species across the different sample groups by stacked bar plots. Each colour represents one taxon and is visualised in the order of increasing relative abundance. Overall, the groups were dominated by *Staphylococcus aureus*, *Klebsiella pneumoniae*, *Staphylococcus epidermidis* and different reads of *Prevotella*. The control group appeared to display a more homogenous bacterial composition, primarily dominated by *Staphylococc*i and *Klebsiella pneumonia*. In the different disease groups with IPF, sarcoidosis and unclassifiable ILD, there tended to be a broader variety of bacterial species, particularly in the middle and lower relative abundance ranges. *Cutibacterium acnes* seemed more abundant in the control and sarcoidosis group compared to in IPF and unclassifiable ILD. The visual impression was that IPF and unclassifiable ILD tended to share similar bacterial profiles, although some species like *Prevotella jejuni* appeared to be more prevalent in IPF compared to in patients with unclassifiable ILD.

### Differential abundance

**Fig. 4:**
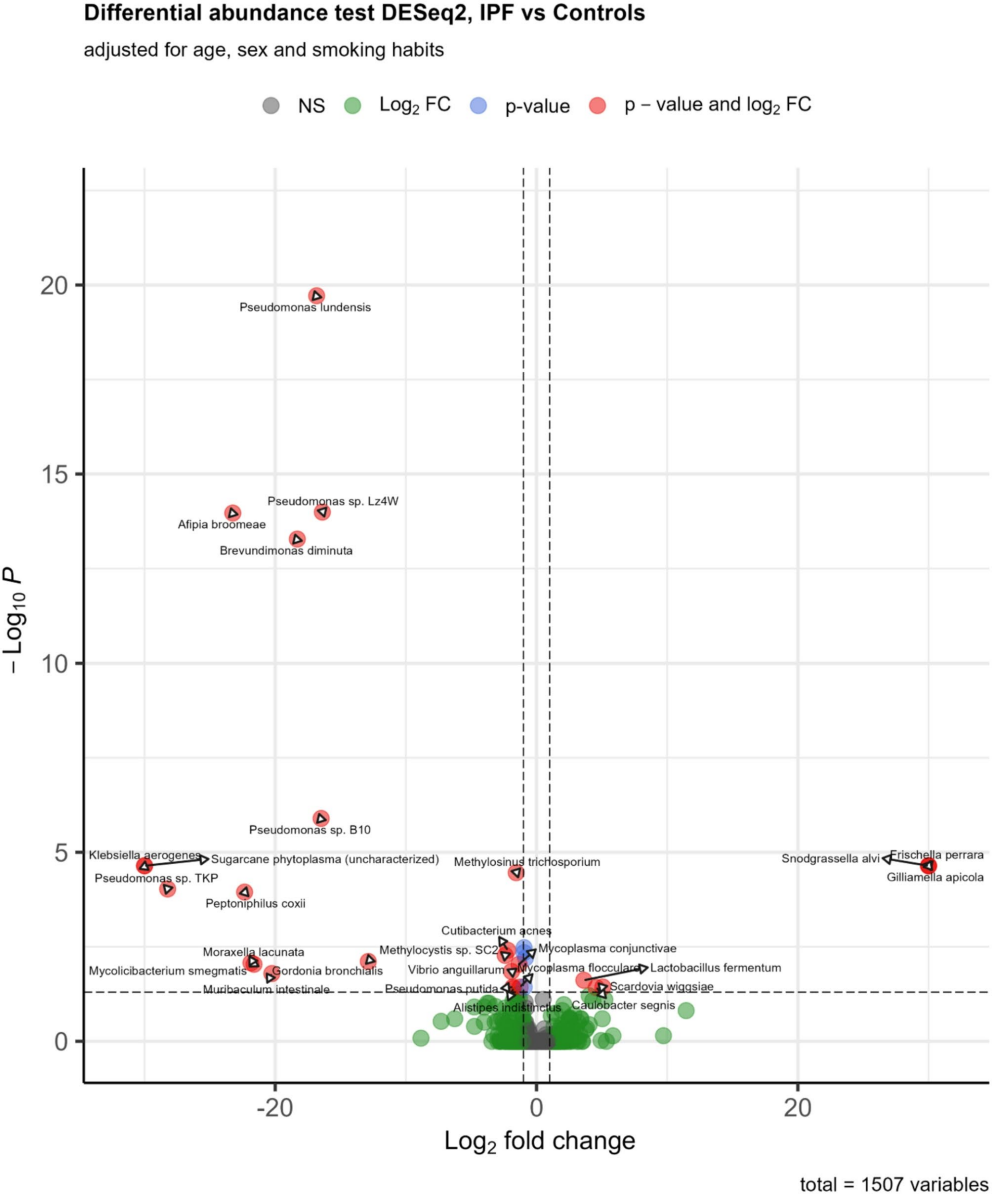
Volcano plot of IPF vs. control species whose abundance were decreased versus the comparison group, are located to the left of zero on the x-axis, while species whose abundance were increased, are located to the right of zero on the x-axis. The dashed vertical line represents the filtering criteria (p-value >-log (0.05)). Every red dot on the plot represents differentially abundant species with increased significance noted towards the top of the plot.Grey dot: not significant

Differential abundance analyses were performed using DESeq2. Pairwise comparisons between study groups of significantly abundant species were visualised with volcano plots. The volcano plot with the comparison between IPF and healthy controls based on the DESEq2 analysis is shown in Fig. 4. The plots comparing sarcoidosis and unclassifiable ILD to healthy controls are provided in the supplementary materials (Supplementary Figs. 1 and 2).

With DESeq2, 32 species were identified as differentially abundant between IPF and controls, 64 species between sarcoidosis and controls, and 28 species between unclassifiable ILD and controls. A complete list of all differentially abundant species is provided in the supplementary materials (Supplementary Table 4). Most of the identified differentially abundant species were low abundant. *Cutibacterium acnes* was significantly less common in all three patient groups compared with controls (*p* < 0.001 for all three comparisons).

### Dysbiosis index

**Fig. 5:**
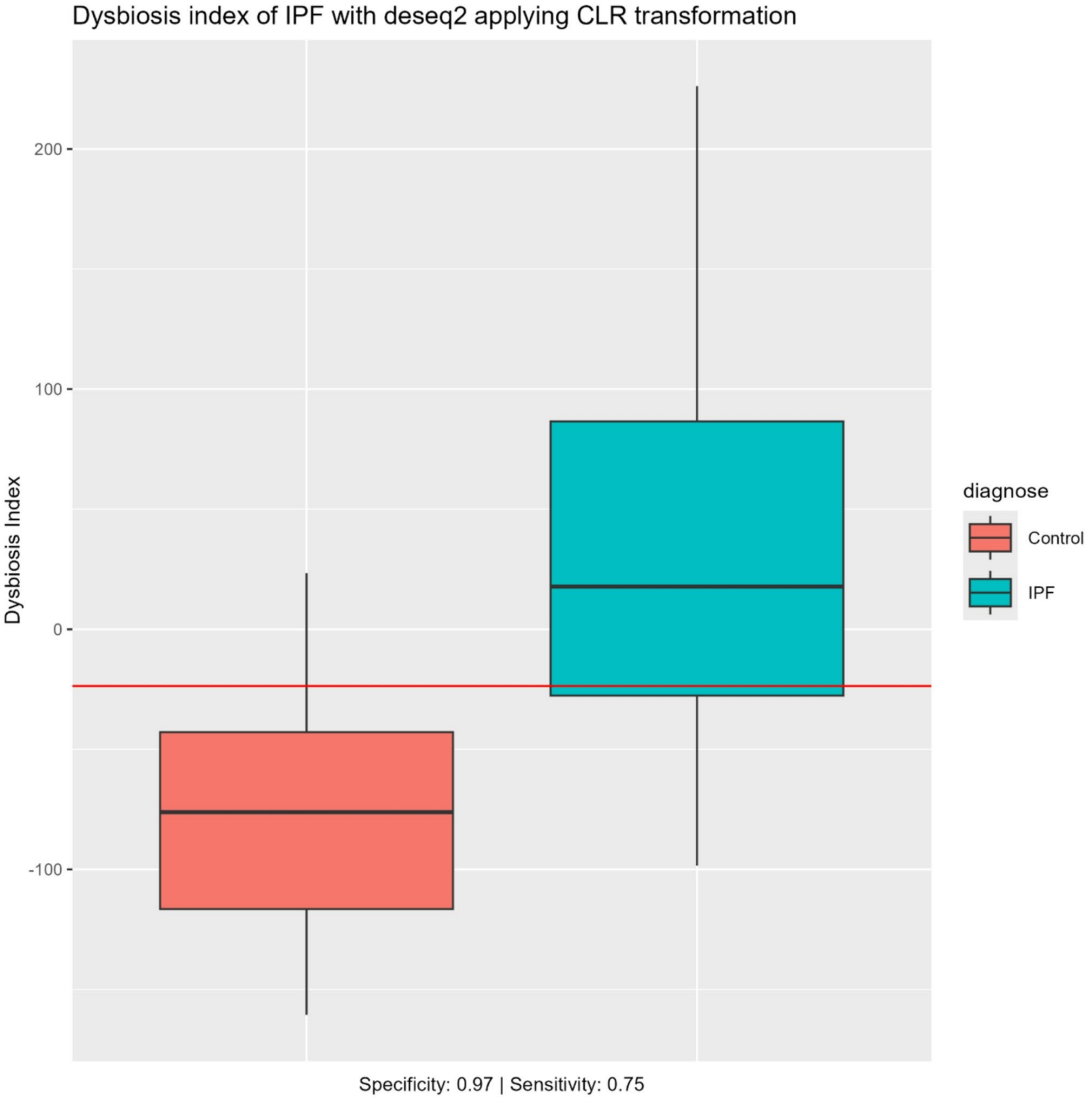
Dysbiosis index of IPF Dysbiosis index (DI) comparing IPF vs. controls. Based on DESeq2 applying CLR transformation. The DI quantifies deviations from the control microbiome, with higher values indicating greater microbial imbalance. The box plot shows the median and interquartile range for each group. The red line indicates significant difference between the groups tested

**Fig. 6:**
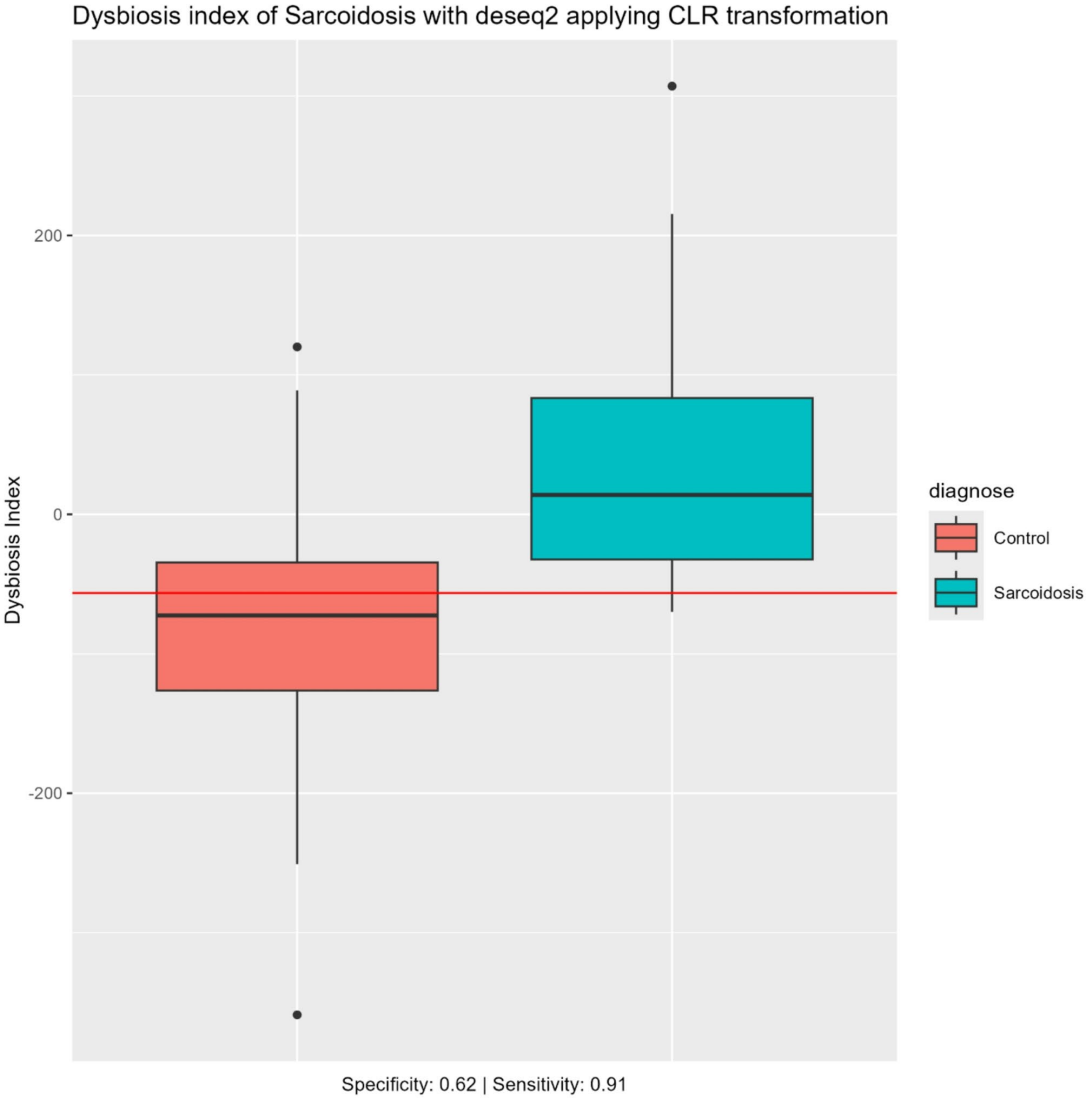
Dysbiosis index of Sarcoidosis Dysbiosis index (DI) comparing sarcoidosis vs. controls. Based on DESeq2 applying CLR transformation. The DI quantifies deviations from the control microbiome, with higher values indicating greater microbial imbalance. The box plot shows the median and interquartile range for each group. The red line indicates significant difference between the groups tested

Figures 5 and 6 illustrates the dysbiosis indices for IPF and sarcoidosis respectively. A horizontal red line in the boxplot indicates the optimal cut point, visually separating the two groups. Employing the dysbiosis index on any given sample, the sensitivity was 0.75 and the specificity was 0.97 for differentiating IPF from controls (Fig. 5). Similarly, for differentiating sarcoidosis from controls the sensitivity was 0.92 and the specificity 0.62 (Fig. 6). By using the optimal dysbiosis index threshold, we observed a clear differentiation between IPF patients and healthy controls.

Of particular interest was to see if we could use the DI to better classify the unclassifiable ILDs. Table 2 illustrates the classification of the 11 different patients in the unclassifiable ILD group according to the dysbiosis index-based threshold values for IPF and sarcoidosis. By applying indices to the samples from unclassifiable ILD patients, we could see that 10 patients were inconsistent with IPF, whereas 8 patients had a potential similarity with sarcoidosis.

**Table 2:** Classification of 11 unclassifiable ILD patients according to dysbiosis index of IPF and sarcoidosis

Patient #	Threshold for IPF	Threshold for sarcoidosis	DI IPF	DI sarcoidosis	IPF?	Sarcoidosis?
1	−23.6	−56.4	−66.01376	29.66105	FALSE	TRUE
2	−23.6	−56.4	−100.06547	99.41851	FALSE	TRUE
3	−23.6	−56.4	−43.57591	66.53463	FALSE	TRUE
4	−23.6	−56.4	−41.51319	−37.33794	FALSE	TRUE
5	−23.6	−56.4	−115.98905	−48.52497	FALSE	TRUE
6	−23.6	−56.4	−94.06089	−117.68660	FALSE	FALSE
7	−23.6	−56.4	−68.70525	−72.67810	FALSE	FALSE
8	−23.6	−56.4	−32.60143	10.79112	FALSE	TRUE
9	−23.6	−56.4	−50.00711	−45.23583	FALSE	TRUE
10	−23.6	−56.4	32.54180	16.34756	TRUE	TRUE
11	−23.6	−56.4	−52.53109	−77.29426	FALSE	FALSE

*DI *Dysbiosis index, *IPF* Idiopathic pulmonary fibrosis

We also examined whether the unclassifiable ILDs aligned better with either sarcoidosis or IPF when looking at subtypes of either disease. By dividing the unclassifiable ILD group into patients with and without ground glass opacities (GGO) and the sarcoidosis group by Scadding stage, the unclassifiable ILDs with GGO appeared to align better with sarcoidosis than unclassifiable ILDs without GGO, with either Scadding stage (Supplementary Fig. 3). Against IPF, dividing the IPF group by FVC lower or higher than 70% of predicted still left us with unclassifiable ILDs having a DI different from most all IPF patients (Supplementary Fig. 4).

## Discussion

This study demonstrates that whole genome sequencing (WGS) can be used to profile the pulmonary microbiome at the species level in both subjects without lung disease and in patients with different interstitial lung diseases. In our study cohort, the unclassifiable ILD patients had significantly different beta diversity and taxonomic profile compared to a healthy control group. The dysbiosis index, calculated from differences in the species level microbiome, is introduced as a potential biomarker for differentiating between interstitial lung diseases.

The human microbiome has emerged as a significant contributor in regulating health and disease [45, 46]. Studies of the bacterial pulmonary microbiome have until recently favored the use of 16 S rRNA sequencing analysis. The disadvantages of 16 S amplicon sequencing are recognized, as this method is less accurate at the species level and sequences only a single region of the genome. WGS holds major advantages as it can sequence broader regions of the genome, providing a more accurate determination at species level and with an enhanced gene prediction [26–29].

Despite the current knowledge about WGS, few studies have evaluated its application in pulmonary microbiome research, likely due to challenges posed by high cost, low biomass and contamination concerns [27]. To the best of our knowledge, no published studies have used WGS to differentiate ILDs based on pulmonary microbiome studies. Furthermore, only a limited number of studies have employed WGS to investigate the pulmonary microbiome [30–36]. Hauser et al. were one of the first to use WGS when analysing sputum samples from two cystic fibrosis patients. They proposed that WGS provides a more comprehensive and accurate qualitative and quantitative assessment of microbiota compositions compared to cultures and PCR [31]. Further investigations have identified core microbiota in lung cancer patients [33], interkingdom mycobiome networks in acute exacerbations of cystic fibrosis [34], and dysbiosis in both critically ill patients [30, 36] and in IPF patients [35]; highlighting distinct microbiome alterations in each group.

Diversity measurements are an essential component of microbiome analysis. In earlier investigations utilizing 16 S rRNA analysis on the same material, we observed a decrease in bacterial alpha diversity among patients with IPF and sarcoidosis as opposed to healthy controls [15, 20]. In contrast, the current study revealed a trend towards higher alpha diversity in IPF and sarcoidosis relative to controls. This discrepancy may reflect methodological differences between 16 S rRNA and metagenomic sequencing approaches, including reduced amplification bias and broader taxonomic resolution with WGS The absence of significant differences in alpha diversity between IPF, sarcoidosis and unclassifiable ILD suggests a similarity in microbial diversity within these disease groups. Similar observations have been reported in previous 16 S rRNA studies [47–49].

The beta diversity was illustrated with a PCoA plot and showed differences among the groups tested. This separation suggests that IPF and the unclassifiable ILD groups had a distinct microbial profile compared to the sarcoidosis and control groups, highlighting potential differences in the microbial composition. Statistical analysis confirmed significantly different beta diversity between the control group and the other groups tested, reinforcing the hypothesis that microbiome alterations may be associated with disease pathogenesis. Despite these differences, the overlap between IPF and unclassifiable ILD in the PCoA plot suggests a shared microbial signature, which may reflect underlying similarities in disease mechanisms or host-microbiome interactions. Further studies incorporating functional metagenomic analysis could help clarify the role of these microbial differences in disease progression.

In this study, the bacterial taxonomic distribution at species level revealed that *Staphylococcus aureus*, *Klebsiella pneumonia*, *Staphylococcus epidermidis* and different reads of *Prevotella* dominated the four groups tested. These taxonomic entities have been identified as being correlated with chronic respiratory diseases affecting the lungs [14, 15, 20, 38, 50]. The high proportion of *Staphylococcus* observed was a somewhat unexpected finding. The genome of *Staphylococcus* shares high sequence similarity with other bacterial species, which can lead to cross mapping, where sequences are incorrectly assigned to *Staphylococcus* instead of their origin source. In contrast, our WGS analysis revealed a lower abundance of *Streptococcus* compared to the levels detected through 16 S rRNA analysis [15, 20]. This discrepancy may be a result of an overestimation of *Streptococcus* abundance in the 16 S rRNA data, for instance due to the typically higher number of ribosomes found in *Streptococcus* [51, 52]. Pienkowska et al. identified *S. aureus* as highly present in cystic fibrosis patients, which can be due to lifelong infections and altering of the microbiome [32]. *Cutibacterium acnes* is known as a member of skin microbiota predominantly found in regions rich in sebaceous glands but is also an opportunistic pathogen [53]. *C. acnes* has previously been associated with sarcoidosis, however its potential causal role remains to be elucidated [54]. *C. Acnes* was visualised as one of the more abundant in sarcoidosis patients and controls, however it was less common in all three patient groups compared with controls. We could argue that typical skin commensals are susceptible to being contaminants in the lower airways. Nevertheless, we addressed this concern by conducting a study using protected sampling, and during the bioinformatics analysis, we filtered out single-read sequences. We should note that our sarcoidosis patients were in a stable state, and taxa being less abundant could theoretically also be a sign that the immune system had been extra vigilant towards *C.acnes*.

Assessing the degree of dysbiosis in microbial samples can provide relevant information to the characterization of the groups studied. Different approaches for utilizing the dysbiosis index are thoroughly described in the review article by Wei et al. ^23^. Defining dysbiosis is challenging due to the substantial inter individual variation observed within the healthy population. To our knowledge, the dysbiosis index has not yet been described in pulmonary microbiome studies. For a dysbiosis index to be most effective, species level characterization of the microbiome is preferable. In this study, differentially abundant (DA) species between each disease and healthy controls were identified with differential abundance analyses, and based on these species, a dysbiosis index was calculated for IPF and sarcoidosis [44]. The index demonstrated elevated sensitivity and specificity when applied to the groups with IPF and sarcoidosis. Subsequently, we used the microbial dysbiosis index to aid the classification of the different patients in the unclassifiable ILD group. The 11 patients in this cohort were categorized in proximity to either IPF or sarcoidosis based on the specific index they exhibited. According to the threshold values, patients with unclassifiable ILD demonstrated greater similarity to both IPF and sarcoidosis patients than to the healthy control group. Further sub-analyses showed that by dividing the sarcoidosis group by Scadding stage, the unclassifiable ILD patients appeared to align better with Scadding stage II and III than with Scadding stage I. And by dividing the unclassifiable ILD patients into those with and without GGO it appeared that unclassifiable ILD patients with GGO had a dysbiosis index more similar to sarcoidosis. This may reflect the predominantly inflammatory nature of having either GGO in unclassifiable ILD or Scadding stage II and III in sarcoidosis, in contrast to the more fibrotic features typically observed in IPF. The dysbiosis index can provide valuable insights, but it is not a diagnostic tool on its own and should be interpreted in the context of clinical data. Future improvements also include longitudinal monitoring over time, which can provide insights into disease progression and treatment response.

Some limitations must be addressed in this study. First, as a cross-sectional study, it is essential to follow up with longitudinal studies to track microbiota changes over time and evaluate their implications for disease development. This is particularly important when identifying pathogenic microbes associated with specific diseases. Second, sampling and quantifying low-biomass microbiota from the lower airways presents significant challenges. The bacterial DNA density in the upper airways is at least 100 times higher than in the lower airways, raising concerns about contamination [55]. To mitigate contamination from the upper airways, we implemented protected sampling and ensured a consistent methodology within a single-center study, with the same staff throughout the entire study period [15, 20, 37]. Additionally, distinguishing bacterial DNA from human DNA remains difficult. The DNA extraction, sequencing, annotation and binning steps are all susceptible to biases [28]. We sequenced 30 negative control samples of sterile phosphate-buffered saline (PBS) fluid not having entered a patient, using otherwise identical laboratory protocols and reagents, without detecting microbial sequences. This suggests that any potential contamination is likely due to bioinformatics misclassification or cross-sample contamination. Our WGS samples had a high level of human contamination, which was removed prior to analysis. As a result, the number of classified bacterial reads was low, potentially contributing to the observed reduction in bacterial diversity and making it more challenging to detect differences between patient groups. Third, this is an exploratory analyses of the microbiome as a potential disease defining biomarker. Ideally, a biomarker should be easy to use, and not overly expensive. Currently that is not the case for the pulmonary microbiome, but technology is advancing rapidly, and the cost of sequencing may decrease further over time. Finally, several sarcoidosis patients had limited disease activity at the time of sampling, reflected by a long interval from diagnosis and a history of prior treatment. This should be considered when interpreting the microbial findings in this group.

To the best of our knowledge, this is the first study employing WGS in profiling the pulmonary microbiome in different ILDs and to categorize them using the microbial dysbiosis index. Given that the majority of ILDs are chronic respiratory diseases with significant global impact on morbidity and mortality, ongoing investigation of microbiota changes associated with these conditions is highly important. Consistent methodologies and robust databases will enhance reproducibility, facilitate meta-analysis and drive advancements in understanding the microbiome. With the continued progress in sequencing technologies, WGS has become more accessible and less expensive. Consequently, this work can be a valuable reference for future studies that highlight microbiome dysbiosis as a key factor in health and disease.

## Supplementary Information

Additional File 1: Supplementary Figure 1. Volcano plot of Sarcoidosis vs Control Species whose abundance were decreased versus the comparison group, are located to the left of zero on the x-axis, while species whose abundance were increased, are located to the right of zero on the x-axis. The dashed vertical line represents the filtering criteria). Every red dot on the plot represents differentially abundant species with increased significance noted towards the top of the plot. Grey dot: not significant

Additional File 2: Supplementary Figure 2. Volcano plot of Unclassifiable ILD vs Control Species whose abundance were decreased versus the comparison group, are located to the left of zero on the x-axis, while species whose abundance were increased, are located to the right of zero on the x-axis. The dashed vertical line represents the filtering criteria (p-value >-log (0.05)). Every red dot on the plot represents differentially abundant species with increased significance noted towards the top of the plot. Grey dot: not significant

Additional File 3: Supplementary Figure 3. Dysbiosis index in sarcoidosis and unclassifiable ILD stratified by Scadding stage and ground glass opacities. Boxplots showing the distribution of the dysbiosis index (DI) in sarcoidosis and unclassifiable ILD patients. Sarcoidosis patients were stratified by Scadding stage (Stage I vs Stage II+), and unclassifiable ILD patients were divided by the presence or absence of ground glass opacities (GGO). The DI was calculated based on differentially abundant taxa identified with DESeq2, using controls as reference

Additional File 4: Supplementary Figure 4. Dysbiosis index in IPF stratified by lung function. Boxplots showing the dysbiosis index (DI) in idiopathic pulmonary fibrosis (IPF) patients stratified by baseline forced vital capacity (FVC ≥70% vs FVC <70% predicted) and unclassifiable ILD patients. The DI was calculated based on differentially abundant taxa identified with DESeq2, using controls as reference

Additional File 5: Supplementary Table 1. A comparison of the prevalence (%) of certain comorbidities and some medications

Additional File 6: Supplementary Table 2. Characteristics of the sarcoidosis patients

Additional File 7: Supplementary Table 3. Characteristics of the unclassifiable ILD patients

Additional File 8: Supplementary Table 4. DESeq2 identified differentially abundant species

## Data Availability

The datasets supporting the conclusions of this article are available in the DRYAD repository, as “Private for peer review”, http://datadryad.org/share/tYl-Sbu18f5UqIWzFyrR8T7WzJGHJQy8va6VApsJ9HU.
