# Supplementary figures and images for: Whole genome sequencing of the pulmonary microbiome in interstitial lung disease subtypes
Source: Respir Res. 2025 Nov 19;26:324. doi: 10.1186/s12931-025-03404-5 (PMC12628550; doi:10.1186/s12931-025-03404-5)

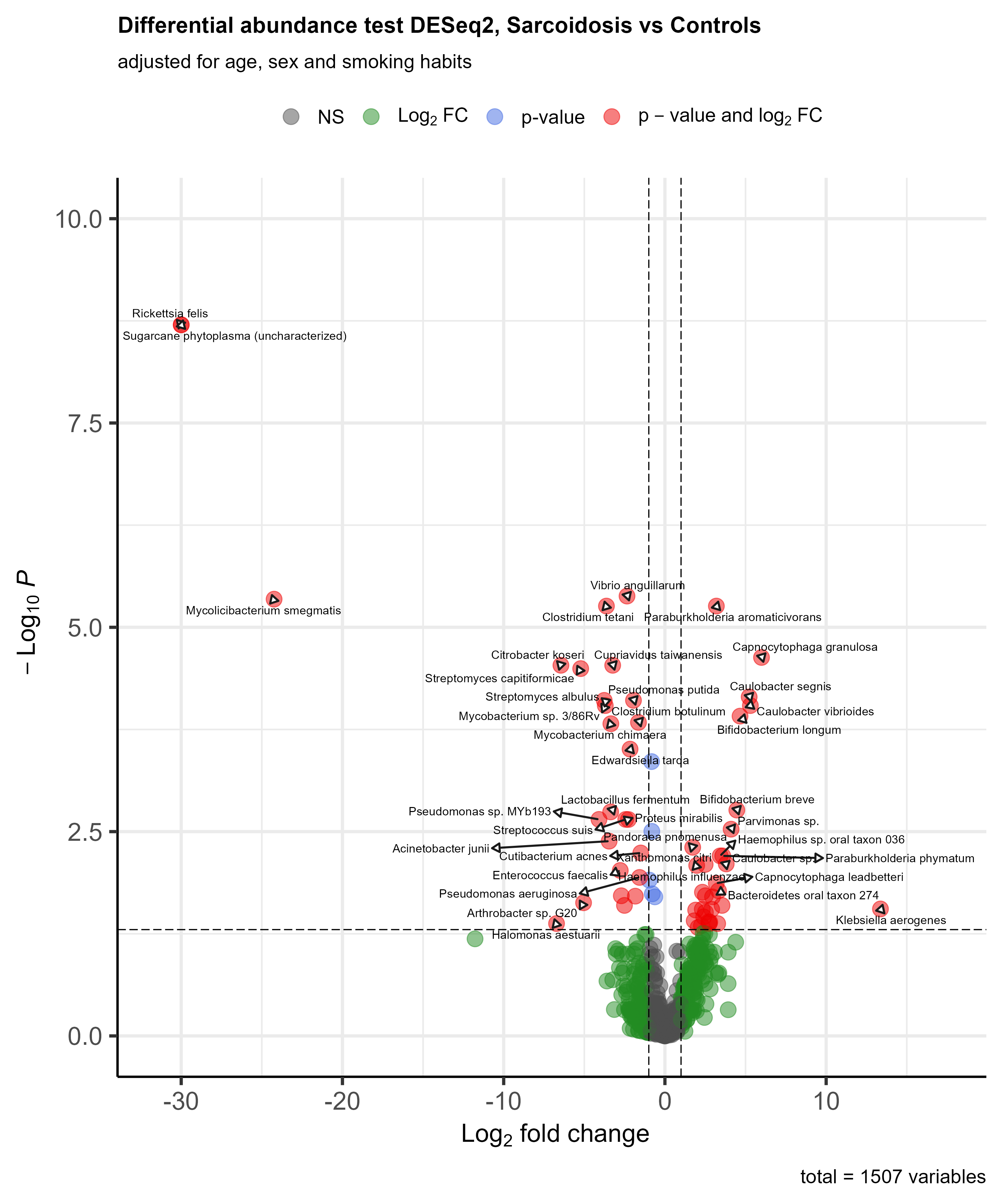

Supplement: Supplementary file 1 — Additional File 1: Supplementary Figure 1. Volcano plot of Sarcoidosis vs Control Species whose abundance were decreased versus the comparison group, are located to the left of zero on the x-axis, while species whose abundance were increased, are located to the right of zero on the x-axis. The dashed vertical line represents the filtering criteria). Every red dot on the plot represents differentially abundant species with increased significance noted towards the top of the plot. Grey dot: not significant [file 12931_2025_3404_MOESM1_ESM.png]

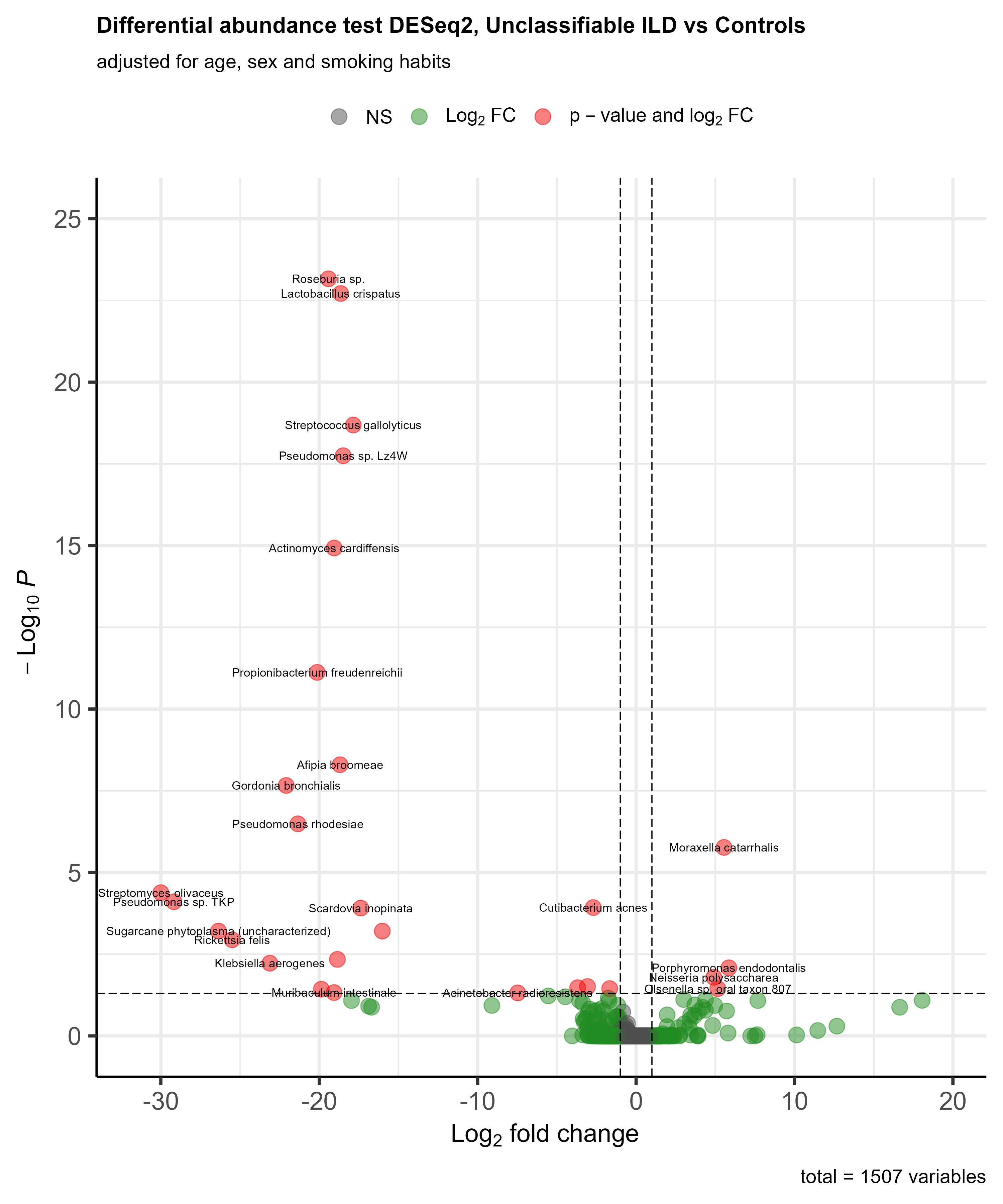

Supplement: Supplementary file 2 — Additional File 2: Supplementary Figure 2. Volcano plot of Unclassifiable ILD vs Control Species whose abundance were decreased versus the comparison group, are located to the left of zero on the x-axis, while species whose abundance were increased, are located to the right of zero on the x-axis. The dashed vertical line represents the filtering criteria (p-value >-log (0.05)). Every red dot on the plot represents differentially abundant species with increased significance noted towards the top of the plot. Grey dot: not significant [file 12931_2025_3404_MOESM2_ESM.png]

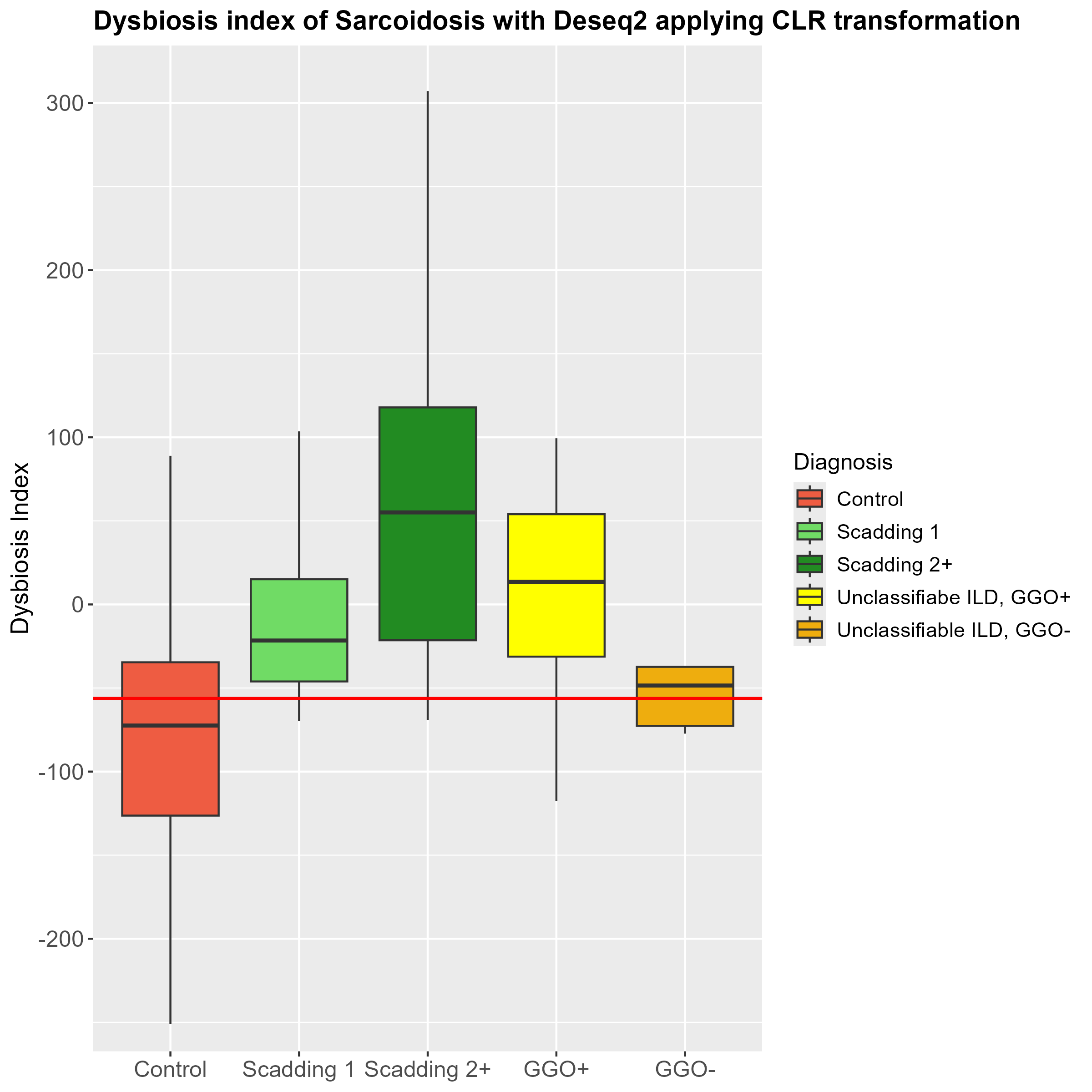

Supplement: Supplementary file 3 — Additional File 3: Supplementary Figure 3. Dysbiosis index in sarcoidosis and unclassifiable ILD stratified by Scadding stage and ground glass opacities. Boxplots showing the distribution of the dysbiosis index (DI) in sarcoidosis and unclassifiable ILD patients. Sarcoidosis patients were stratified by Scadding stage (Stage I vs Stage II+), and unclassifiable ILD patients were divided by the presence or absence of ground glass opacities (GGO). The DI was calculated based on differentially abundant taxa identified with DESeq2, using controls as reference [file 12931_2025_3404_MOESM3_ESM.png]

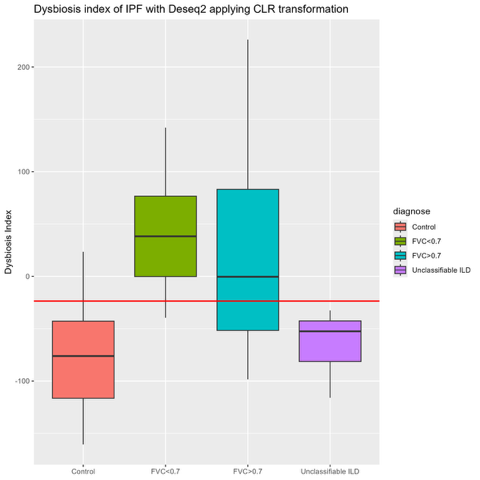

Supplement: Supplementary file 4 — Additional File 4: Supplementary Figure 4. Dysbiosis index in IPF stratified by lung function. Boxplots showing the dysbiosis index (DI) in idiopathic pulmonary fibrosis (IPF) patients stratified by baseline forced vital capacity (FVC ≥70% vs FVC <70% predicted) and unclassifiable ILD patients. The DI was calculated based on differentially abundant taxa identified with DESeq2, using controls as reference [file 12931_2025_3404_MOESM4_ESM.png]
